# Local Places Ruling Life: Compromises and Restricted Career Choices in Rural Sweden

**DOI:** 10.1007/s43151-022-00085-5

**Published:** 2022-11-03

**Authors:** Elin Ennerberg, Janna Lundberg, Malin Axelsson

**Affiliations:** Department for Society, Culture and Identity, Malmö University, Malmö, Sweden

**Keywords:** Place, Choice, Career, Work, Education, Youth

## Abstract

In this article, we explore the theme of choice in relation to education and work by discussing the notion of “compromise” within career guidance. In particular, we analyse compromises related to career choice in regard to groups and individuals who are restricted — or belong — to a particular geographical place and how this spatial limitation is managed and discussed by career guidance counsellors who work with unemployed individuals. The article is based on the following questions: How are compromises and restricted choices considered in relation to place and belonging? How do career guidance counsellors and other professionals discuss compromises of choice? The qualitative interviews have been conducted with career counsellors working with unemployed individuals in rural Sweden. Four themes emerge: location as a given/non-issue, location as opposed to interest, location as personal life and location as an obstacle. We argue that belonging can be seen as more available to certain groups. For example, career counsellors view the claims of belonging by young people as less valid than for individuals who can present caring and family obligations as reasons for not being mobile.

## Introduction

Choosing a career can be seen not only as a decision that determines one’s professional life but also as a choice that reflects or rejects a certain identity. In reality, career choice is not necessarily a one-time event as contemporary working life can necessitate multiple career changes. Moreover, though the imperative of choice accompanies the area of career guidance, many researchers have pointed to the lack of real choice that confronts many individuals (see for example Roberts 2009). Nonetheless, the imperative of “choosing” carries the strong societal expectation not only of choosing employment for financial reasons of self-sufficiency but also of expressing a certain interest or using certain skills that suit the particular individual. In this article, we explore the theme of choice by discussing the notion of “compromise” in relation to career guidance. In particular, we discuss compromises related to career choice in relation to groups and individuals who are restricted — or belong — to a particular geographical place and how this spatial limitation is managed and discussed by career guidance counsellors and other professionals who work with unemployed individuals. Our conclusion states the importance of spatial connections and belonging. Not being able to move is presented as problematic by the case workers, but belonging can also be seen as an important resource, rather than as an obstacle to creating a certain career.

The research questions guiding this paper are constructed as follows:

- *How are compromises and restricted choices considered in relation to place and belonging?*
- *How do career guidance counsellors and other professionals discuss compromises of choice?*

Our study reflects two different themes related to career guidance that have been explored by other researchers. First, discussions of place/space have emerged as an important research area to understand both labour market changes and structural consequences of industrialisation processes for rural communities and individuals’ decision-making and thoughts in relation to moving from a particular location to work or study. Secondly, this literature relates to research on individual constraints in relation to social mobility and opportunity structures. The perspective of how career guidance professionals consider issues of mobility and belonging is under-researched in rural sociology. In this study, we aim to fill this gap in particular by considering career guidance professionals’ own agency and dilemmas in regard to counselling unemployed individuals in rural settings.

In this article, we begin by reviewing previous research. First studies on individual choice related to studies on space, place and locality are presented. We also highlight how individual career choice is understood in certain career theories, which are frequently used by career counsellors in meetings with clients. We then introduce our theoretical framework, where career theories are discussed in relation to the sociological theory of “personal lives” (Smart 2007; May and Nordqvist 2019) and theories of belonging (Aaltonen 2021; Cuervo and Wyn 2014, 2017; Harris et al. 2021). The interview methodology for our study is described and followed by a presentation of our findings and analysis. We have carried out 13 interviews over eight months. After analysis, they have resulted in four accounts of location: location as a given/nonissue, location as opposed to interest, location as personal life and location as an obstacle. Our conclusion in the discussion sums up location in contrast to ideas of mobility, belonging and choice in the guidance counsellors’ and case workers’ conceptions of their work. Finally, we present the local embeddedness, belonging and compromises of ongoing life at a specific local place and work as a way to challenge and question the mobility imperative in mainstream policy.

## Previous Studies — Place and Constraints

Choice in relation to career and education is often framed as an act of decision-making that focuses on finding a vocation that best suits the individual’s needs and interests. In these discourses, spatiality may play a role, but mainly as one of many factors that individuals need to take into account when making their decisions. However, many recent studies focusing on space and locality in relation to career and education opportunities have highlighted geographical location and belonging as central in understanding choice.

Labour market and educational opportunities often impact individuals’ sense of whether they see themselves staying or moving away from a place, particularly in rural areas. Studies focusing on who leaves the countryside generally find that young women move to a greater extent than young men (Karpestam and Gladoić Håkansson 2021), often not only to go to greater city regions where more feminised work is available, but also to pursue higher education (Johansson 2021). Reasons for leaving are, however, often framed by respondents as an active choice — related to, for example, exploring new opportunities rather than being forced to move for labour market reasons (Boström and Dahlin 2018). The idea of a “cosmopolitan” identity and the cultural expectations of participating in urban life as an important part of youth culture can be seen as shaping individuals’ aspirations to be mobile (Skrbis et al. 2014). Regarding mobility, the research on elite schools presents a contrasting field since they are often discussed as places where a cosmopolitan mobility habitus is fostered. Elite schools teach their students to move between similar elite institutions globally, within a “geography of privilege” (Zanten et al. 2015). In contrast, rural youth have a more ambivalent view of mobility. This sense of ambivalence is referred to by rural students interviewed by Rönnlund (2020) with the phrase “I love this place, but I won’t stay” (p. 123). Rönnlund explains this as “a feeling of being torn between a desire to stay and pressure to leave” (p. 131). To really commit to achieving self-realisation, the students need to leave, but their identity and who they really are is still connected to the rural area of their origin (Rönnlund 2020).

Corbett (2007) argues that the imperative “learning to leave” influences children and youth already at school in a coastal community in Canada. Similarly, Uddbäck (2021) claims that a “mobility imperative” influences young Swedes in a small town when they come to terms with their decision to stay. For those who stay regardless of the mobility norm, they construct their identities in relation to the place in terms of a specific “place habitus” and a strong work ethic whereby they manage to find work in local industries. Aaltonen (2021) also finds a strong sense of belonging among individuals in rural Finland, where the motivations for staying relate both to material and emotional factors. Although work in this region was scarce, many individuals tried to utilise local opportunities, such as rural entrepreneurship, as a way to manage to stay in the region. Harris et al. (2021) point out how the label of un-belonging has been used to portray vulnerable groups. Similar to the results in this study they show that belonging can be strong even when individuals can be found in a vulnerable position. They also emphasise belonging as something beyond just a “good thing”. Belonging can be problematic and something good at the same time, for those who are seen as un-belonging, at-risk or stuck (Harris et al. 2021). In our analysis, we show how belonging to a place, a locality, can be understood as a strength and a weakness and as a compromising choice mechanism.

Lack of labour market opportunities for young individuals in rural regions can also be seen in Wenham (2020), where the youth interviewed experience “multiple forms of deprivation”, e.g. unemployment coupled with incomplete schooling and difficult family situations. The idea of leaving for higher education or labour market opportunities was here often seen not only as too daunting but also as difficult due to complex family and caring responsibilities. These youth also experienced their employment opportunities as very limited and had few future plans for work or education. Similarly, individuals at a different costal town in the UK described the difficulties of finding stable work locally; however, they did not see moving away from the area or commuting for work as an option easily available due to high living and transportation costs (Reid and Westergaard 2017). Issues of spatiality in most previous research are intimately connected to constrains in relation to class and to educational background.

Fjellman (2019) shows that Swedish students in Upper Secondary school level at rural settings have fewer educational programs to choose from than to students in urban settings. Relatedly, Rosvall et al. (2018) argue that rural students are to a greater extent than urban students influenced by economic constraints when choosing upper secondary education programs, if limited choice is available locally, as moving in order to study requires additional financial means. In this sense, choice based on interest is primarily available to rural students from more privileged economic backgrounds or students in urban areas. Rosvall also (2020) analyses school counsellors’ work in rural locations in relation to students’ choice and local space. Here, students’ desires to stay and their local place attachment are often downplayed in favour of narratives that promote educational and work opportunities, often portrayed as necessitating mobility. Alexander and Hooley (2017) similarly underline that local labour market and educational structures influence graduate students’ choices in remote coastal regions in the UK, where individuals feel the expectations of leaving, while simultaneously expressing a strong local connection to the place where they grew up. The researchers also emphasise that career counsellors here face a dual role, where regional and national labour market needs have to be considered and balanced against the individuals’ own circumstances.

The roles of career counsellors and their professional commitments are highlighted in the *NICE Foundation Handbook*. While labour market support is underlined as the reason for career counselling being fundamental in a changing society where lifelong learning has become more prescient, many of the ethical and professional guidelines emphasise support for the individual as the central theme (Schiersmann et al 2012), perhaps implying that the needs of the individual should be put before those of the labour market in the professional meeting.

## Theoretical Framework

Compromise in relation to career choice is discussed in different career theories. Gottfredson (1996) sees compromise in relation to career decisions as an event that takes place when individuals need to adjust their preferred choice, often because of external circumstances. As a prior process, Gottfredson also describes circumscription, where individuals’ own preferences are modified in order to make a choice that is seen as a more acceptable option in relation to gender stereotypes. The compromise is thus achieved when individuals first have rejected certain options and then settle for and modify their occupational choice to achieve an acceptable alternative. Another career theory that deals with the notion of compromise is found in Holland (1997), where individuals’ personality traits are discussed in relation to educational and vocational options. For example, compromise is taken as needed if an individual’s own attributes are seen as incompatible with the characteristics needed in a specific employment. Inherent in these career theories is the idea that individuals generally have a clear and strong original preference for a specific career. This career choice seems to spring from the individual’s own self, but may or may not be adapted both due to societal obstacles (e.g. an adaption to contemporary gender norms) and labour market constraints (e.g. having to take a job in a less desirable sector or at a lower level).

More recent career theories, such as from Savickas and colleagues (Savickas et al. 2009), emphasise “life design” as a way to acknowledge individuals’ various life circumstances and how these can impact on careers. However, both the idea of individuals having particularly clear ideas of their own career choice and the idea of individuals planning and “designing” their lives seem to match the contemporary “cult of the individual”, whereby individuals are seen as particular, unique beings who often craft their identities in relation to career choices (cf. Beck and Beck-Gernsheim 2002).

As Hayes (cited in Cuervo and Wyn 2014) points out, the literature on “transitions” also implicitly supports the idea of the individual moving to new opportunities, whereby work and education are singled out as particularly important and defining for the individual. To complement and question this idea of work as central to individual identity, Cuervo and Wyn (2014, 2017) use the metaphor of “belonging”, not only to highlight the significance of individuals’ lives beyond the labour market and particular moments of transition, but also to add a spatial dimension to individuals’ lives. Belonging as a concept is used to analyse other parts of individuals’ grounding by emphasising the importance of personal relationships, both in terms of understanding individuals’ career decisions and how their sense of belonging is constructed and grounded through these relationships. For example, Smart (2007) argues that individuals acquire an embeddedness connected to a certain place through the “sticky relationships” that have an impact on individual practices and give individuals an ontological security (2007, p. 45). Instead of emphasising “individualisation” as a main guiding factor in individuals’ lives, empirical studies show that relationality is an important aspect of individual decision-making (Carter and Duncan 2017). May and Nordqvist argue further that “it is important to understand both the personal and the social in order to examine not only personal life but also society” (May and Nordqvist 2019, p. 177). This would indicate that career choices not only are influenced by individualised pursuits, but that they may also be constructed in relation to the roots connected to different localities.

In this article, we use the concepts compromise and belonging together in order to discuss how career choices can be restricted by location. The different concepts can be seen as tools to discuss critically some of the assumptions in career choice and guidance.

## Methodology

The material in this study was collected over a period of 8 months. Our aim was to interview a range of professionals who worked with unemployed individuals or individuals considering a career change in small towns and rural areas in a local Swedish region. In total, 13 interviews were conducted. The professionals interviewed mainly had a career guidance education background. Although two of the interviewees had a different educational background, they worked in professions associated with career guidance, where they had acquired similar competencies.

The local region where we have conducted our interviews is characterised by a number of smaller towns most of which are not only in close proximity to the coast but also encompassing a relatively sparsely populated forested areas 10–50 km from the coast. Closer to the coastal towns, the country side is witnessing gentrification and becoming less affordable for local residents in terms of housing. The area has a number of rural villages and smaller hamlets which are within relatively close proximity to smaller towns where education and labour market opportunities are accessible. Employment is often available in local industries but also within the hospitality, retail and service sectors as well as the public sector in health care and education. Though a rural setting, agriculture represents a smaller share of the labour market sector, providing work for less than 3% of the regional population (Arbetsförmedlingen 2021). The region also has a reputation for supporting entrepreneurs and a growing tourism sector. Educational opportunities are available through a local university in the region, specialising many educational opportunities with a strong labour market connection. Furthermore, vocational higher education is provided by various actors in the region. Local municipalities also offer shorter vocational training courses for unemployed individuals.

Five interviewees worked with labour market support, either at the local public employment service or at the local municipality, and eight interviewees worked at the local municipality adult education guidance services. The caseworkers’ tasks often varied depending on which client group they were meeting. For example, one case worker at the local labour market office mainly met clients who in need of more extensive help and support. Other professionals working with adult education guidance services mainly met clients in need of help finding information about education or work. In this sense, the material can be seen as limited as it covers a range of experiences rather than focusing on similar work routines. However, the broad experiences of working with different clients that we encountered in the interviews have facilitated a broader and more nuanced understanding of the different types of issues and work that encompass meeting unemployed individuals in a local labour market.

Informants were recruited through local contacts at the municipalities and through the authors’ own professional networks. Due to the Covid-19 restrictions, interviews were held through video tools, where the sound from the interviews was recorded and subsequently transcribed verbatim. Qualitative interviews are useful for gathering information from informants on a specific topic and allowing for follow-up questions (Kvale 2007). Interviews can also be seen as informal talk where the respondents’ ideas are constructed together with the interviewer’s. Digital interviews have the advantage of being relatively easy to conduct if informants have access to the digital tools. This proved to be the case in our sample, with many informants finding the time for the interviews relatively quickly. A disadvantage with online interviewing is that less space is given to informal small talk and making informants feel comfortable with the interview situation (O’Connor and Madge 2017, p. 425). In our study, it seemed as if most respondents were happy to delve right into the topic of the interview, perhaps as they were mainly talking about aspects related to their work. All informants were given written and oral information about voluntary participation; how the material was to be gathered, managed and stored; and of the opportunity to withdraw their consent at any time. As some of the informants may know one another due to local connections and working in a relatively small geographical area, we chose not to mention the municipality in which they work. Moreover, we do not include their specific work title when we refer to them in this article, although we occasionally mention the more general work place if this is needed to contextualise the data. In this way, we have taken extra steps to ensure internal anonymity of the sample. In the research process, we have taken into account the advisory guide of good research practice as formulated by the Swedish Research Council (2017).

The interview material was coded in Nvivo and analysed abductively, as the researchers moved back and forth between theoretical concepts and the empirical material. Through reading and re-reading the material, certain themes emerged. These themes were discussed in relation to the research questions and previous studies in the area. When the citations were translated from Swedish to English, a number were slightly edited to increase clarity for the reader.

One of the limitations of the study is that we only base our analysis on the guidance counsellors/caseworkers’ stories. That is, the information we are given regarding individuals’ choices is necessarily a secondary source of information, where the informants may give us a narrative that is shaped by their own values, organisational identity and other possible aspects. Our empirical data and analysis cannot claim any certainty with regard to how the individuals they refer to in their accounts actually feel or act. Regardless of these limitations, the interviews have allowed us to gather material for answering our research questions, with the focus on the career counsellors/caseworkers’ accounts regarding choices and compromises related to space and career guidance practices.

## Findings and Analysis

In the findings, we consider how the caseworkers/guidance counsellors discuss location and belonging from different perspectives when relating their work with clients.

### Location as a Given/Non-issue

One of the ways location is discussed by caseworkers is by referring to location as a given, non-negotiable circumstance for the clients they meet. For certain clients, different vulnerabilities in relation to, for example, unemployment, housing and health issues make it difficult to contemplate a move for work:

So, if you look at our clients, most of the time it’s not possible for them to pack up and move, because they can’t get a new housing contract anywhere if they don’t have an income. They receive municipal social assistance. So… applying for work in a different location is a bit complicated since you don’t have anywhere to live when you arrive in a new place. (Informant B)

This above indicates some of the issues involved with regard to re-locating for individuals who simply do not need to think about whether they would like to move to find a job that suits their interests. Instead, for individuals who are dependent on local assistance, private ties such as housing become a particularly strong obstacle to mobility. Apart from the necessity of having housing financed in the local municipality where the individuals live, other aspects also influence the lack of mobility for individuals with particular vulnerabilities:

But it’s the others who are not so willing to relocate. Those are the individuals we have to work most with, and it is this group who receive sick insurance and who need rehabilitation and work assessments before they are even able to work part-time. (Informant C)

For some of the clients discussed by this career counsellor, being on sick leave or in need of rehabilitation measures also necessitate staying locally. Rather than considering mobility as a way to explore work opportunities elsewhere, locally directed support is seen as necessary in order to first build up a capacity to work. The location of the individuals is therefore seen as a given because of all the central ties of the support network the individual is dependent on are situated locally:

Yes, part of it is to be able to feel secure. And then some individuals not only have contact with the social services but also with other medical contacts related to psychiatric care, and then they can get a bit worried about losing those contacts. And of course, that’s understandable. And a different aspect is also maybe that one feels a sense of security related to friends, and when you’re in a bad place […] then you’re not really ready to leave ... (Informant D)

As this quote shows, the local network that is important for individuals can also include a complex web of services that are not directly related to the labour market, but that still make mobility seem a non-issue. A move to find work in a different location is here discussed in terms of a very complex decision, one which only seems available to individuals who have a certain minimum of resources. For this group, however, locality and place are seen as a given. And when meeting career counsellors, career choices for the individual are in this way necessarily considered in relation to local education and the labour market.

### Location as Opposed to Interests

A second way location is discussed by our interviewees is when it is portrayed as opposing interests. The idea that career interests should be the starting point for making a decision, as indicated by career theorists such as Gottfredson (1996) and Holland (1997), can be seen in certain accounts by caseworkers/guidance counsellors:

And maybe you could add that, because maybe that’s one aspect that sort of frustrates me, that clients don’t choose the education that they would really like. Rather, they just consider ‘These are the only educational courses in City X and what is then the best of the courses that are closest to where I live? (Informant H)

This narrative shows a normative idea that ideally career choices should be prioritised over location. The idea of starting with the location and considering which educational opportunities is available locally can thus be considered as a less optimal alternative, as the most interesting or suitable career may thus be abandoned if not available locally. Individuals prioritising location over career ambitions can therefore be seen as making a suboptimal choice. Another informant not only considers this from a slightly more neutral perspective, but also sets location against a “dream job” as a potential clash:

Yes, so it’s above all that then they have to be aware that it will have consequences for the choices they can make. […] It’s the choices between Ok, I want to go for my… well you can call it my dream, or maybe my plan is a better word. […] but then I have to make some kind of move. […] Or if I make the choice to stay, that might be plan A or Plan B and what does that choice entail? So that you also make a conscious choice. But Ok, if it’s important for me to stay, then I have to make my choices based on that decision. And in that case, there is one type of labour market here locally. (Informant E)

In this citation, we can clearly see that the compromise the career counsellor discusses is in line with that as understood by certain career theorists (Gottfredson 1996; Holland 1997). When the idea of a certain dream career is the primary goal of individuals’ lives but staying within the local labour market is an impediment, individuals either have to adjust or to compromise. In the next citation, the career counsellor not only sets up a similar dilemma, but also considers how guiding in a neutral way needs to be considered in relation to local circumstances:

I’m usually very careful in explaining that I am happy to have the interview and that we can discuss different aspects. But as a career and guidance counsellor, it is also important for me not to guide you towards unemployment. So, we also have a discussion of “what do these choices lead to? And how can you utilize this? And how do we reach your vocational goals and not only educational goals through going down this path?’ And there you can end up having very interesting discussions. Then I would never discourage someone from following their dreams, but it’s more that we include [that discussion] in the interview as well. (Informant F)

In meetings with clients, the local space versus the dream career are discussed as two different potential routes. If the location is seen as being prioritised, this is emphasised in the conversation as leading to potentially suboptimal career choices, where the local career market will be the starting point for the career guidance process. Emphasising the local labour market and envisaging the potential limits can be seen as tools used to visualise this potential clash of interests, as illustrated later on in the interview with the career counsellor:

If I’m aware that the labour market is very limited for a certain career, then I usually leave it up to the client and ask if they know about the labour market demands and if they can tell me more about their plans. And many don’t know. And we talk about how they can find more information, or other times they might realise in their story that the dream will be difficult to follow if they stay where they are. And then some will take the decision not to follow their dream, whereas others reflect upon this and then make the decision to move. It depends on what has the biggest pull and what aspects are most important. But those meetings can be a bit challenging. Because the greater your limitations, the harder it is. (Informant F)

In the above accounts, two kinds of frames of discussing work can be seen among the career counsellors. On the one hand, career choice is seen as a normatively better starting point from which to plan and guide individuals towards. On the other hand, many of the informants also strive to maintain a position of neutrality in the meetings to avoid steering individuals towards certain specified routes. This can be seen as a reflection of their professional roles and competencies (as exemplified in NICE guidelines), but the different accounts also show the dual role of combining individual support with a labour market perspective (cf. Alexander and Hooley 2017).

### Location as Personal Life

A third way to discuss location is in relation to personal life commitments that influence individuals’ career decisions. In these accounts, individuals’ struggles or vulnerabilities are not emphasised. Rather, the emphasis is on their more positive or strong personal place attachments. One informant specifically discusses the geographical location as essential for building a future career in this location:

Many clients say ‘I don’t want to leave the coast, I don’t want to leave my friends, I’ve grown up close to the sea’. And often when they’ve considered their options, it’s also in relation to still wanting to live by the sea. And I think that’s a bit exciting because it’s been a recurring theme […] that they have a strong connection to their geographical location: ‘I’ve grown up by the sea, I always want to be close to the sea and to be able to smell the sea’ and similar sentiments. Not really connected to surfing, or liking swimming or anything like that. And sometimes I think it’s that they think it’s similar to home, even away from home. (Informant F)

Place here is described as specifically framed among the individuals. It is embedded with special meaning in relation to the natural environment offered at the location, which is seen as a very particular sense of belonging (cf. Rönnlund 2020). In one sense, moving to a similar location (also close to the sea) is seen as a potential possibility, if a move is necessary. However, the quote also encompasses both the feeling of “home” as important and the less developed theme of staying close to friendship ties in this particular location. A similar discussion emphasises individual roots and belonging in relation to family:

Well, mostly this is expressed by adults, because they have a family, a house and all that close by and then they don’t want to… then they don’t have the opportunity to move because of studies or anything. But then they often ask whether there’s any remote courses available. And also, you might not want to let go of a job, but at the same time you might need to study first to be able to complete your previous education […]. We’ve just started an industrial education here for both adults and youth. And then also adults who have been outside of the labour market for a while have applied to it to be able to potentially get a job in a local industry here. (Informant G).

For the individuals described here, the significance of being older, established in terms of family life and material commitments, such as housing, constitutes a strong commitment to place. This sense of acknowledging roots and belonging can be related to the personal life perspective (May and Nordqvist 2019; Smart 2007). Interestingly, belonging is also potentially more acceptable specifically due to family life commitments. Rather than simply “not wanting to move”, the respondent changes the description to “not finding it possible to move”. Here, belonging becomes associated with a long-time commitment. Moreover, as individuals have more strongly confirmed their roots through previous life choices, this seems to make it more difficult for career counsellors to counter with a mobility narrative. Belonging is in this way potentially less available for the young, whose commitments are potentially seen as less valid arguments:

Then it’s these other clients who are young and who might just have… they have a girlfriend or they play football, or well it’s something small that means it’s still nice and safe to stay. They are a bit easier to challenge some way and start looking at other places and seeing opportunities with different choices. You can use yourself as an example, that I [studied in various locations], and it sort of broadens [their horizons], and it’s really exciting to see those opportunities. […] That they can see that [leaving] isn’t necessarily so bad. (Informant H)

Another respondent also discusses young people’s choices in terms of staying and leaving by highlighting “But I still think that as a human being you should take the opportunity to see the world when you’re between 18–30” (Informant M). Accordingly, young people also seem prone to claiming belonging to a local place in terms of social interests or personal commitments through intimate relationships. Unlike the other quote, these commitments are not seen as equally valid; rather, they are placed against an expressed mobility norm that emphasises widening horizons and the excitement of moving as part of what can be expected from a young person (cf. Skrbis et al. 2014).

### Location as an Obstacle

The final theme in relation to location is the portrayal of location as an obstacle to entering the labour market. For some of our informants, a strong local connection is argued as being a problem in terms of limiting individuals too closely to the local job market.

Well, it can be that you’re a member of some association, and perhaps you’re happy with your social life. You have certain commitments there that you could compare to a position the working life. But it’s through third sector associations, and you’re very content with that. And it’s also quite common that unemployed individuals get a dog [laughs], so they’re very busy for large portions of the day [laughing]. So, I’ve also met many who say ´Yes, but I’ve got my dog …’ ‘Well yes you have your dog, but many people who work also have dogs’. So you sort of have to build it [into the conversation] that there are dog day-care facilities, and so on. (Informant C)

The many local and place bound commitments for individuals who need to enter the labour market are here constructed as being problematic in terms of thinking more broadly about work and education. To have a strong sense of belonging to a place becomes a difficulty from the case worker point of view. For one informant, this is particularly valid for individuals with a lower educational background:

And I think that people with a higher education background are also willing to commute in order to do what they want, so low education can be an obstacle from that perspective. To commute for work, to get any type of work, is also a matter of money. The economic difference isn’t that great, but it’s a lot harder. (Informant J)

Individuals with a higher educational background are seen as less place bound and potentially more able to commute for working opportunities, even if they might not necessarily move to a different place. In contrast, commuting for a lower-paid job is often more difficult to motivate; as such, employment does not necessarily render a higher income if performed in a different location. Furthermore, there are the additional time frames of commuting and of the travelling costs involved. The mobility norm is thus stronger in relation to individuals that are seen as having the economic and educational opportunities to move, thereby validating unemployed individuals’ local commitments and embeddedness. Here, a different type of compromises in relation to career choice can potentially be distinguished for different individuals.

Individuals who enter lower educational opportunities are place bound in a different way. Local municipalities can offer vocational labour market education to the unemployed, but generally this should be tailored to the local labour market needs. As one respondent disclosed, “And then it could be that the vocational education that we offer in this municipality is not a vocational education that they’re interested in” (Informant C). In practice, then, higher education can symbolise mobility, whereas local labour market educations tie the individual both to local labour market educational opportunities and thereby directly to the local labour market needs, indicating that many clients here face a more enforced type of belonging (cf. Harris et al. 2021).

## Conclusion

In the previous section, we have discussed location from four different perspectives in order to understand how mobility, belonging and choice are present in the work of guidance counsellors and case workers.

In the narratives, compromises are not simply related to career choices where individuals structure their life decisions around an optimal career. Instead, space and belonging can play a more important role in managing career changes and career choice. The caseworkers and career guidance counsellors to a certain extent reflect both a mobility norm in terms of moving to find work and the idea that individuals’ decisions should be structured around career decisions. On the other hand, neutrality in choice can be used to support individual decision-making that prioritises location. For individuals who have strong family commitments, belonging can also be seen as an acceptable factor for avoiding moving for work. Similarly, individuals with strong needs of additional support due to problems with health or other factors are seen as necessarily locally embedded.

When compromising around current life situations in relation to place and work, the main factor affecting what type of work the guidance counsellors suggest for their clients is their place and (private) life. Individuals in vulnerable positions are not in a position to move. Those who already have a permanent job and can provide for themselves are obviously not in a position — nor need — of mobility unless by choice. The individuals that are the subjects of mobility seem to be the educated middle-class without responsibilities of care (cf. Smart 2007). This result poses interesting questions regarding who is able to claim a local connection, belonging, as necessary in order to stay and build a life in the place that is referred to as home. The mobility imperative seems first and foremost to apply to youth with potential ambitions to work and study, even when individuals in this group also express a strong desire to stay in the place they love.

The mobility imperative — which has been an important policy in terms of encouraging individuals to move and to participate in higher education — also highlights the importance of change as well as to “design” our individual lives in relation to career ambitions. At the same time, individuals’ own lives and stories emphasise belonging as roots and embeddedness. For those finding it necessary to move despite wanting to stay (cf. Rönnlund 2020), mobility to find other work options can be seen as just as an important compromise as staying and focusing on a local career. Emphasising the importance of spatial connections and belonging may, from a different perspective, instead be seen as important resources, rather than as obstacles to creating a certain career. While labour market policies focus on mobility and flexibility, in practice, local conditions strongly influence individuals; and caseworkers and career counsellors adjust their advice to both local and individual circumstances. While challenging the mobility imperative, this also creates potential opportunities in terms of considering the local labour market needs when helping individuals choose careers. For those living in rural parts of Sweden, the choices are fewer (e.g. Fjellman 2019). Thereby, the people living in rural parts make choices adapted to the possibilities available. However, adapting to the choices may also enable individuals to make career choices that are grounded in their personal lives as well as the local circumstances.
